# Non-contrast T1 mapping characterizes the myocardium beyond that achieved by late gadolinium enhancement in both hypertrophic and dilated cardiomyopathy

**DOI:** 10.1186/1532-429X-14-S1-O27

**Published:** 2012-02-01

**Authors:** Sairia Dass, Joseph Suttie, Stefan K Piechnik, Vanessa Ferreira, Cameron Holloway, Matthew D Robson, Hugh Watkins, Theodoros Karamitsos, Stefan Neubauer

**Affiliations:** 1 Deprtment of Cardiovascular Medicine, University of Oxford, Oxford, UK; 2 Deprtment of Physiology, Anatomy and Genetics, University of Oxford, Oxford, UK

## Summary

T1 relaxation is prolonged in hypertrophic (HCM) and dilated cardiomyopathy (DCM) and reflects myocardial changes prior to presence of LGE or development of hypertrophy in HCM and wall thinning in DCM.

## Background

Both HCM and DCM exhibit variable clinical outcomes. Unfortunately, our ability to risk stratify and characterize disease mechanisms in these patients remains poor, and improved biomarkers are needed. Myocardial fibrosis, both focal and diffuse, is a feature of both HCM and DCM and focal myocardial fibrosis, as assessed by LGE, has been recently identified as a predictor of cardiac death in HCM and DCM.

We recently developed a novel method for non-contrast CMR T1 mapping, a recognized marker of increased water content such as in fibrotic tissue. We hypothesized that T1 mapping offers an alternative to late gadolinium enhancement (LGE) techniques, providing additional non- invasive diagnostic information in HCM and DCM.

## Methods

Sixty one subjects underwent CMR imaging, 3T Siemens Trio,(29 HCM, 18 DCM and 14 normals). Matching short axis slices were acquired for cine, T1 mapping (short modified look locker inversion recovery - ShMOLLI - sequence) and late gadolinium enhancement (LGE) imaging (0.1 mmol/kg). Myocardium was manually contoured on each slice and divided into 6 segments to calculate: mean T1, mean LGE enhancement (segment intensity divided by signal in remote myocardium), end diastolic wall thickness from cine images.

## Results

**Figure 1:**
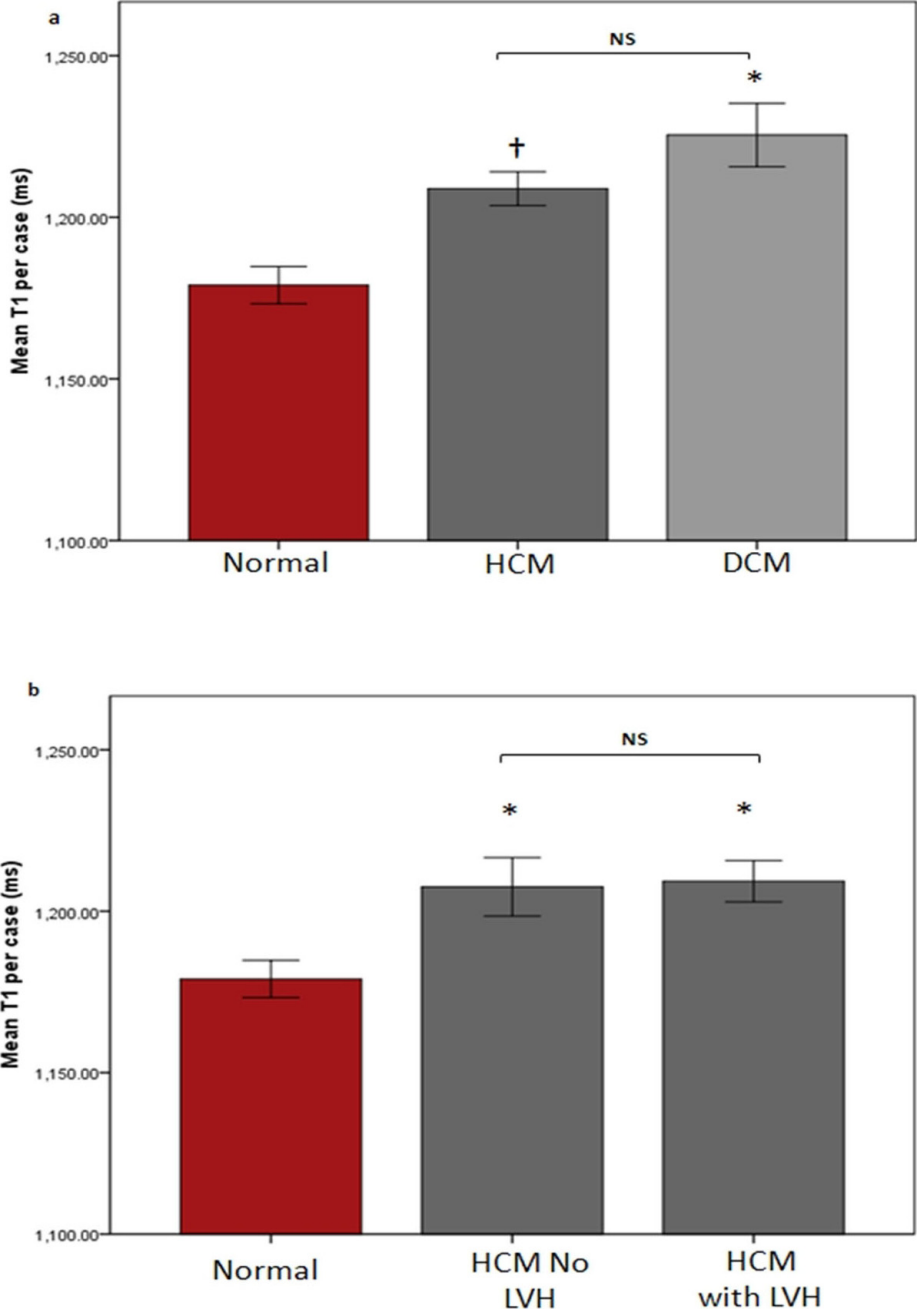
Myocardial T1 relaxation times in a) normal controls, HCM and DCM; b) normal controls, HCM pre hypertrophy and HCM with hypertrophy. P values represent 3 way Bonferroni corrected ANOVA, * represents P<0.0001 vs normal, † P<0.01 vs normal. Error bars are standard error of mean.

**Figure 2:**
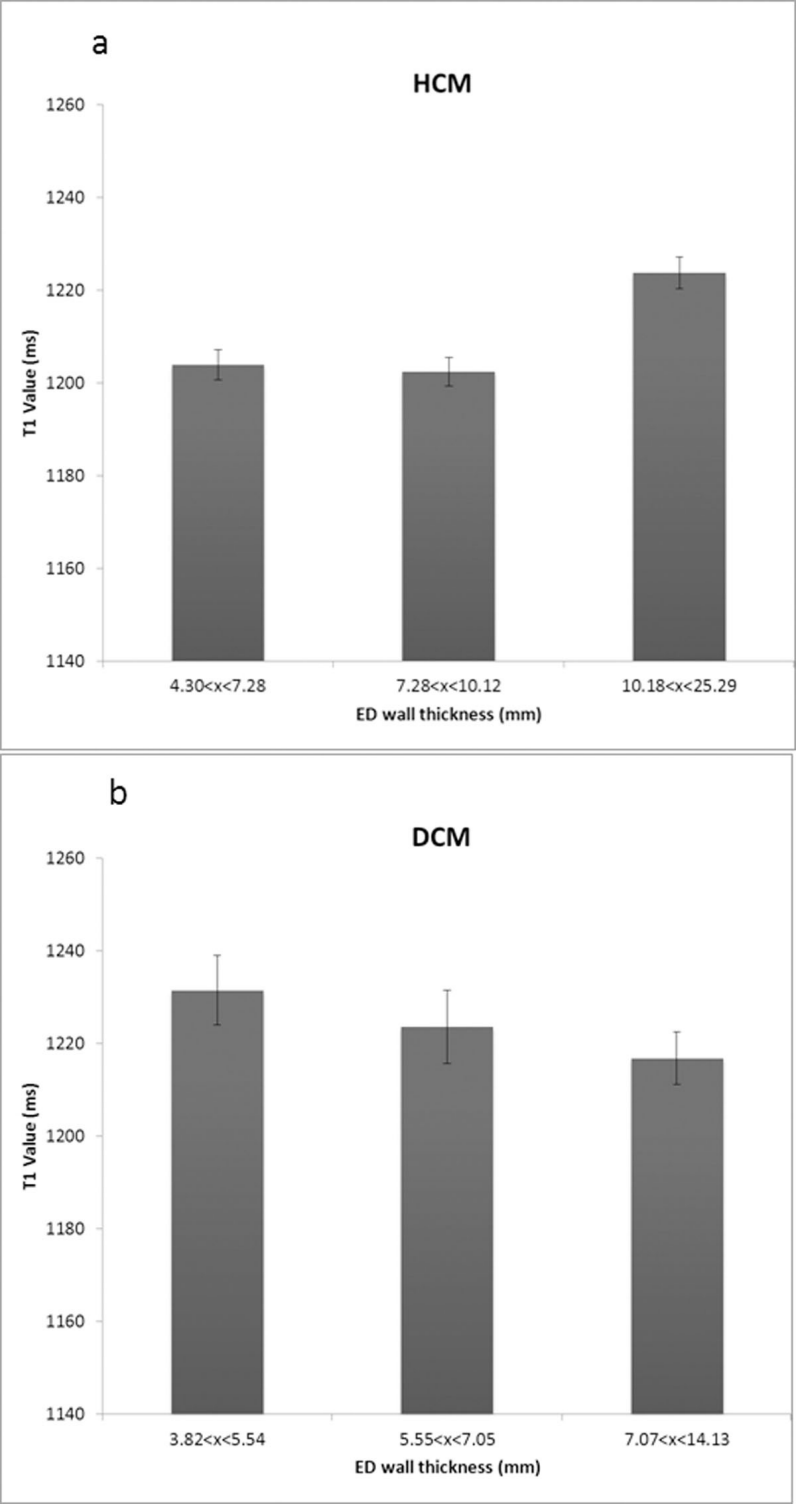
In HCM and DCM, T1 relaxation time ploted in tertiles of end diastolic wall thickness, error bars are standard error of the mean.

Mean T1 relaxation time was significantly elevated in HCM and DCM (HCM 1208±47ms, DCM 1224±69ms, normal 1172±45ms, p<0.05, figure 1a). Interestingly, myocardial T1 values were elevated in HCM gene carriers without LVH (n=8) to similar levels as those with LVH (n=21), figure. 1b. None-the-less T1 was elevated irrespective of wall thickness. However, there was a positive correlation between T1 values and wall thickness in HCM. In DCM, there was a negative correlation, figure 2.

There was a weak, but significant, correlation between mean T1 and LGE (r= 0.32, p< 0.001). T1 values were significantly higher in segments with LGE than in those without (HCM with LGE 1229±42ms vs no LGE 1193±44ms, p<0.05; DCM with LGE 1252±87ms vs no LGE 1213±55ms p<0.05). However, even in segments unaffected by LGE, T1 values were significantly higher than normal (p<0.001).

## Conclusions

In HCM and DCM, non-contrast T1 mapping detects underlying disease processes which to some extent reflect the presence of LGE but yields further insights into adverse ventricular remodeling beyond the traditional measures of wall thickness and LGE. Myocardial T1 values are elevated in HCM patients without LVH and in DCM patients pre wall thinning suggesting that absolute T1 mapping may also have applications in the detection of early myocardial disease. Hence, T1 mapping may become an important new tool for diagnosis, therapy monitoring and prognostic assessment in HCM and DCM.

## Funding

This research was funded by the British heart foundation.

